# Combination of deep XLMS with deep learning reveals an ordered rearrangement and assembly of a major protein component of the vaccinia virion

**DOI:** 10.1128/mbio.01135-23

**Published:** 2023-08-30

**Authors:** Yeva Mirzakhanyan, Andris Jankevics, Richard A. Scheltema, Paul David Gershon

**Affiliations:** 1 Department of Molecular Biology and Biochemistry, University of California Irvine, Irvine, California, USA; 2 Biomolecular Mass Spectrometry and Proteomics, Bijvoet Center for Biomolecular Research and Utrecht Institute for Pharmaceutical Sciences, University of Utrecht, Utrecht, the Netherlands; Columbia University Medical College, New York, New York, USA

**Keywords:** poxvirus, vaccinia virus, structural biology, mass spectrometry, structural proteomics

## Abstract

Vaccinia virus, the prototypical poxvirus and smallpox/monkeypox vaccine, has proven a challenging entity for structural biology, defying many of the approaches leading to molecular and atomic models for other viruses. Via a combination of deep learning and cross-linking mass spectrometry, we have developed an atomic-level model and an integrated processing/assembly pathway for a structural component of the vaccinia virion, protein P4a. Within the pathway, proteolytic separation of the C-terminal P4a-3 segment of P4a triggers a massive conformational rotation within the N-terminal P4a-1 segment that becomes fixed by disulfide-locking while removing a steric block to trimerization of the processing intermediate P4a-1+2. These events trigger the proteolytic separation of P4a-2, allowing the assembly of P4a-1 into a hexagonal lattice that encloses the nascent virion core.

**IMPORTANCE:**

An outstanding problem in the understanding of poxvirus biology is the molecular structure of the mature virion. Via deep learning methods combined with chemical cross-linking mass spectrometry, we have addressed the structure and assembly pathway of P4a, a key poxvirus virion core component.

## INTRODUCTION

Vaccinia, the prototypical member of the poxvirus family, is a large DNA virus with just under 200 genes. It comprised the vaccine used for smallpox eradication and is the current vaccine against monkeypox. On the basis of genomic similarity, all three of these viruses are likely structurally identical. Over the past three decades, substantial insights have been gained into the biology of vaccinia at the functional level: broad roles have been deduced for most of the essential virus genes via phenotypic analysis of natural or engineered conditional mutants (1). The ultrastructure of the vaccinia virion has been apparent in outline since the 1960s, comprising an ellipsoid or “brick-shaped” structure whose core contains the genome with various enzymes and structural proteins, enclosed by at least one lipid envelope with envelope proteins. The core wall is perhaps the virion’s most tangible ultrastructural feature though it likely has substantially less structural rigidity than the capsid of a non-enveloped virus (2 – 5). Atomic structures for 34 of the 75–80 proteins packaged within the mature virion (MV) can be found, currently, in RCSB Protein Data Bank. A much more limited picture has been achieved of the virion’s internal molecular architecture. Reasons for this include the size and complexity of vaccinia, its polymorphic nature and overall asymmetry, and the presence of at least one phospholipid envelope. As an intact entity, vaccinia has largely defied the classical approaches of X-ray crystallography and cryoelectron microscopy (cryoEM)/tomography.

Chemical cross-linking with bifunctional reagents (cross-linking mass spectrometry; XLMS) can inform structural biology in multiple ways, for example, by constraining atomic models of individual proteins, guiding the docking of proteins of known 3D structure, and providing protein molecular interaction network models. In the latter regard, our preliminary XLMS study of vaccinia revealed intra-virion protein-protein networks in outline (6). We have fundamentally extended this XLMS data set, resulting in, after stringent thresholding, a saturating data set comprising ~135,000 cross-link-spectral matches (CSMs) representing ~22,000 unique cross-links (XL) on the basis of protein accession, cross-link position and cross-linked peptide Mr. To assemble these into a molecular model, a desirable starting point would be atomic models for at least the major structural protein components of the virion—the very entities for which classical structural biology has been unsuccessful at providing atomic models, perhaps as a result of their higher-order complexity and intrinsic insolubility.

Recent deep learning approaches for the *de novo* prediction of protein structure have been described as having, to a large extent, solved the “protein folding problem.” Initially skeptical, particularly with regard to vaccinia structural proteins—the majority of which are unique to the poxviridae—we generated structural predictions merely as placeholders for XLMS network analysis. However, via exhaustive statistical and other benchmarking studies, we came to regard some structural models as approaching experimental accuracy (manuscript in preparation). In the study described here, a vaccinia major structural protein, P4a, was structurally modeled via deep learning methods. In combination with experimental data (XLMS), the resulting structural models yield insights into processing, conformational change, disulfide rearrangement, and higher-order assembly to the level of the whole-virion.

## RESULTS

Vaccinia protein P4a is one of the virion’s most abundant protein components. It is conserved in all known poxviridae with no identifiable sequence or structural homologs outside this family. Conditional mutants under nonpermissive conditions exhibit an interruption of normal virion morphogenesis, reduced virus yield and an accumulation in the cytoplasm of abnormal immature virus (IV)-like particles lacking the normal, dumbbell-shaped core morphology (7 – 17). During virion morphogenesis, P4a is proteolytically processed at two sites (at residues 614 and 697) marked by the diamino acid AG| (9, 11, 17 – 23). Processing at these two sites is critical for normal morphogenesis (20, 24 – 29).

### P4a structural models

**Fig 1:**
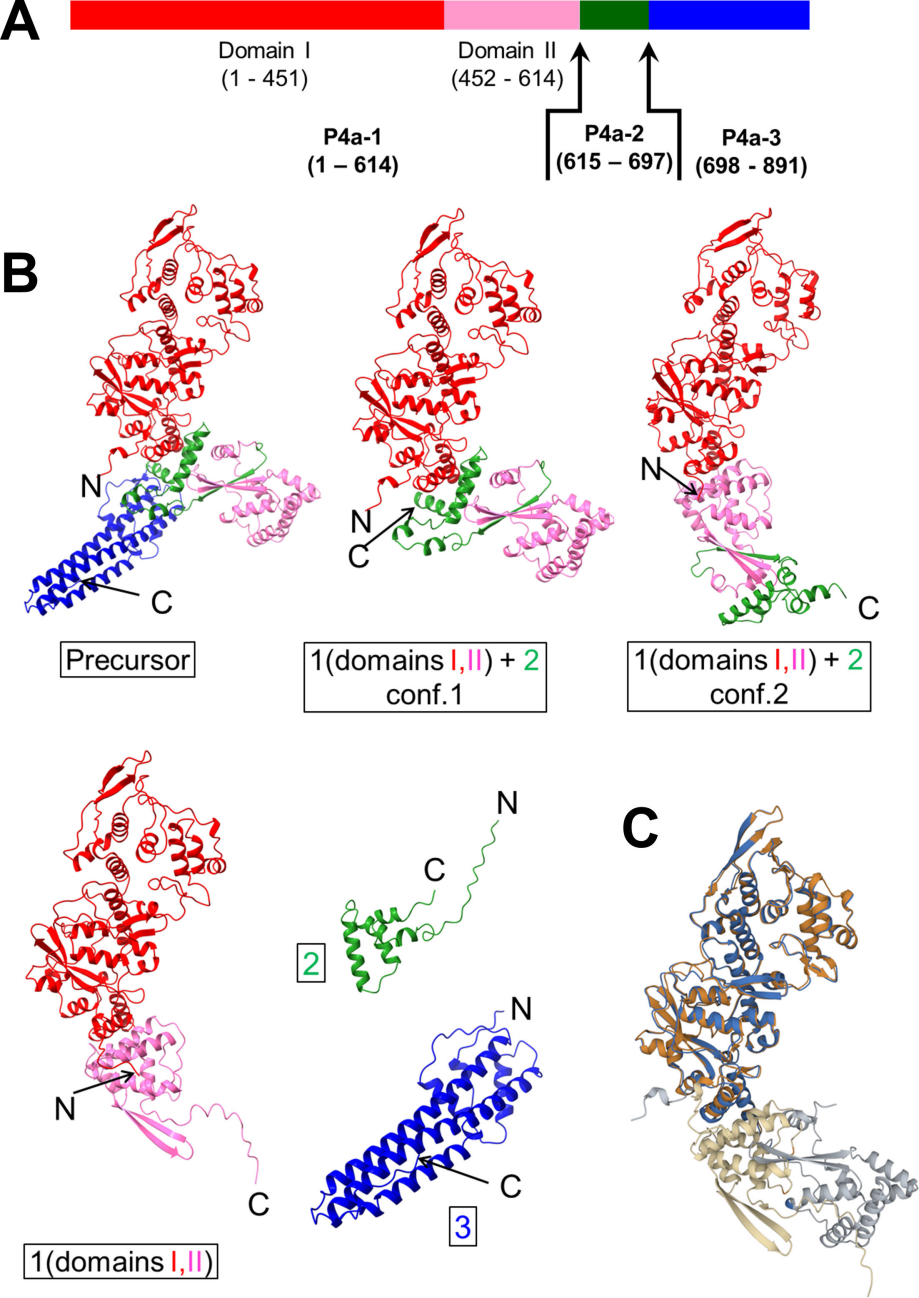
Structural models of P4a. (**A**) P4a precursor as a linear chain, colored by processed segments (P4a-1, P4a-2, and P4a-3) and domains of P4a-1. Black arrows indicate AG| processing sites. (**B**) Structural models for P4a precursor and processing products P4a-1, P4a-2, P4a-3, and the two P4a-1+2 intermediate forms. P4a-1 was predicted with two domains denoted here I (red) and II (pink) connected via a hinge. Green and blue: P4a-2 and P4a-3 segments, respectively. Conformational inversion at the hinge corresponded with the removal of P4a-3, the C-terminal processed product of P4a. (**C**) Aligned AlphaFold2 predictions for the P4a-1 segment from P4a-precursor (residues 1–614; blue: Aligned residues; gray: Residues not in alignment) and P4a-1 processed product (orange: Aligned residues; tan: Residues not in alignment). P4a-1 domain II has rotated ~180°.

The three major products of P4a processing are referred to here, in linear order as P4a-1, P4a-2, and P4a-3 (Fig. 1A). P4a-1 and P4a-3 are packaged, while P4a-2 is likely discarded and eliminated at the proteasome (19). Despite an absence of sequence homologs outside of the poxviridae, atomic structures for P4a-precursor, P4a-1 and P4a-3 could be predicted by AlphaFold2 (Fig. 1B; Fig. S1) with high statistical confidence (Fig. S1). An equally confident structure was predicted for the P4a-1 segment of the presumed cleavage intermediate P4a-1+2 (Fig. 1B; Fig. S1). No significant structural homologs were identified to any of the above forms of P4a (data not shown) supporting the uniqueness of this protein to the poxviruses.

### Conformational hinging

The AlphaFold2 model for P4a-1 showed two subdomains (denoted here as P4a-1 domains I and II; Fig. 1B) connected by a hinge that could support a substantial (~180°) rotation of domain II with respect to domain I. This rotation was evident by the comparison of models for the P4a-1 segment in precursor versus processed forms (Fig. 1C; Movie S1). Supporting the authentic modeling of both conformations, precursor and product models of the P4a-1 segment showed equally strong average pLDDT scores (Fig. S1). Models for mature P4a-1 predicted a single conformation exclusively (Fig. S2), and those for the N-terminal region of P4a precursor spanning segments P4a-1 and P4a-2 showed almost exclusively a single conformation (Fig. S2). Models for the P4a-1+2 cleavage intermediate, however, clustered neatly around a pair of distinct conformations which differed at the hinge (Fig. S2; Fig. 1; Fig. S1), strongly suggesting that this intermediate, specifically, was bistable.

The conformation of mature P4a-1 could be verified from experimental intra-protein XLMS data from intact MV: Of the summed XLMS CSM count of 4,009 available for intra-P4a-1 cross-links, a subset totaling 3,829 (95.5% of the overall total) supported the conformation modeled in mature P4a-1 (Fig. S3), while a subset totaling just 2,265 (only 56.5% of the overall total) supported the conformation of P4a-1 in the P4a precursor model (Fig. S4). Among the distance-violating cross-links in the latter model that were structurally rational in mature P4a-1, the majority lay directly across the P4a-1 hinge, strongly supporting the “post-rotation” conformation of P4a-1 in the virion *in vivo*.

### P4a-3 removal as the trigger for conformational hinging

In the precursor conformation, P4a-1 domains I and II are separated from one another by a wedge-like interjection of the P4a-2 segment into the P4a-1 hinge. This interjection comprises the three C-terminal alpha helices of P4a-2 nestled between two helices of P4a-1 domain I and one helix of P4a-1 domain II, along with a three-stranded beta sheet comprising one strand from the N-terminus of P4a-2 and two strands from P4a-1 domain II (Fig. 1B: “Precursor”). This conformation of P4a-2 (and thereby of the overall precursor) seems to be stabilized, externally, by the P4a-3 segment which, while flexible in absolute placement (data not shown), modeled consistently in the vicinity of the hinge in the precursor model (Fig. 1B: “Precursor”) and presents a barrier to P4a-2 movement or rearrangement. Cleavage at P4a’s downstream AG| processing site to release the P4a-3 segment removes the external brace, destabilizing the P4a-2 wedge and allowing hinging to the “post-rotational” P4a-1 product conformation (Fig. 1B; Movie S1).

### Disulfide locking

Models were next scanned for cysteine pairs lying within the potential intra-protein disulfide bonding range. One such pair was found, namely Cys31 and Cys569. These two cysteines (which are conserved in all known poxvirus P4a sequences) are located within the P4a-1 segment, one each in domains I and II where they directly span the hinge. While they lay far outside disulfide bonding range in P4a precursor (Fig. S7) and in the P4a-1+2 intermediate conf.1 (prerotation conformation; data not shown), they are within disulfide bonding range in fully processed P4a-1 (Fig. S7) and in the P4a-1+2 intermediate conf.2 (post-rotation conformation). After AG| processing and removal of the P4a-3 product, a potential mechanism for locking of the post-rotational conformation is provided by the vaccinia-encoded and packaged oxidoreductases (30 – 32), whose activity in disulfide bond stabilization is documented (33).

### P4a-1 trimerization

**Fig 2:**
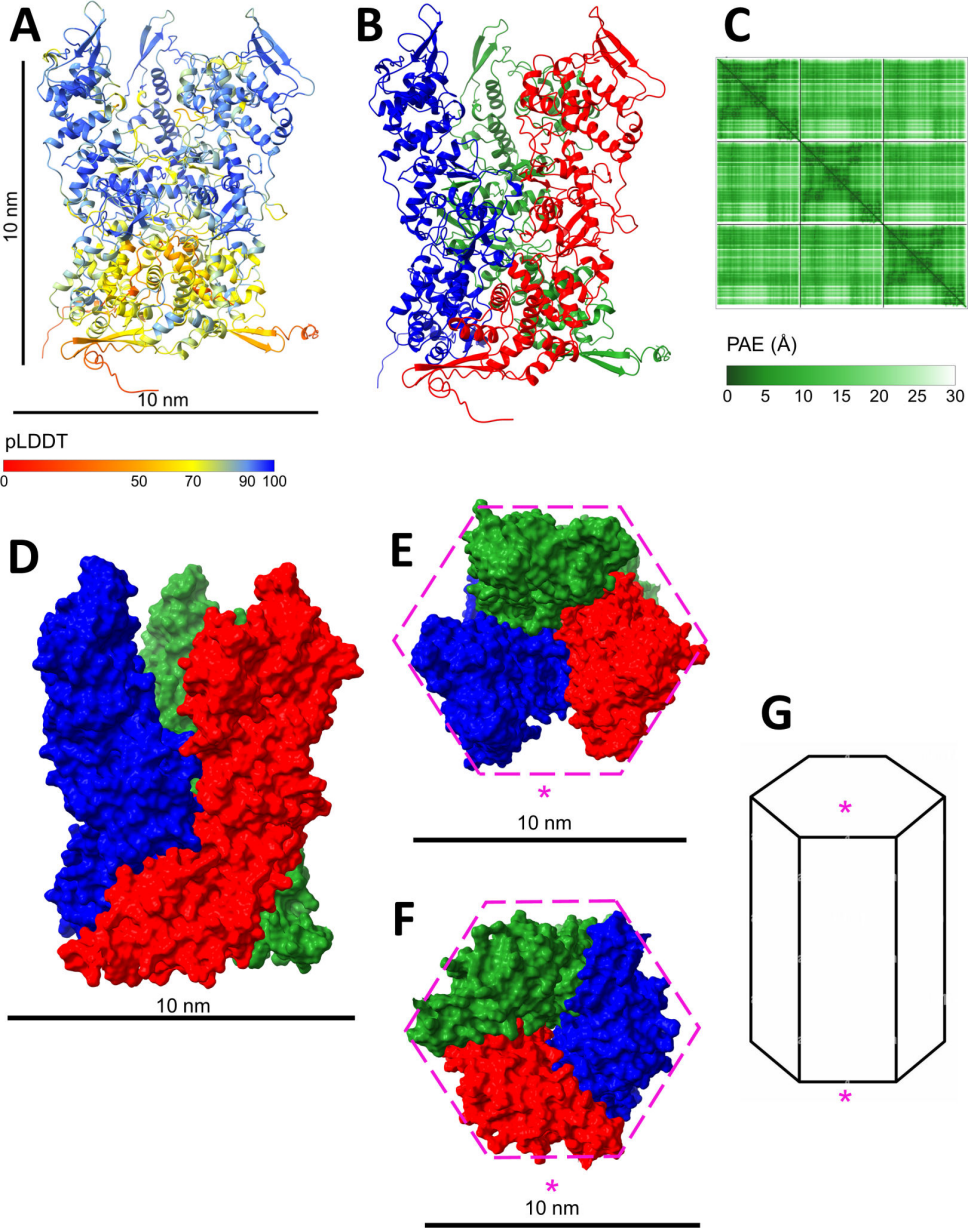
P4a-1 trimer. (**A**) Colored pLDDT (scale bars: 10 nm), (**B**) Colored by P4a-1 chain within the trimer. (**C**) PAE plot for trimer. Color scale is in Angstrom units. (**D–F**) Trimer in spacefill from side, above, and below, respectively (colored as in Fig 2B) showing the mutual enwrapping of subunits at the base, and an outline approximating to hexagonal geometry (residues 600–614 excluded from view). (**G**) The trimer model can be fit within a hexagonal prism geometry (upper and lower hexagons are in phase), with a ~60° twist along the body of each subunit such that the starred side, viewed from above (**E**) corresponds to the starred side viewed from below (**F**).

To investigate the higher-order structure of P4a-1 in MV, structural predictions were attempted for P4a-1 dimer, trimer, tetramer, and pentamer using AlphaFold-multimer. Among these, the trimeric model showed a particularly high confidence (Fig. 2A and B) and by far the strongest PAE plot (Fig. 2C; Fig. S8). Moreover, a very high degree of convergence was noted between the 25 trimer models reported by AlphaFold-multimer (Fig. S9 and S2). The trimer model adopted the approximate shape of a cylindrical “candlestick holder” with “opening petals,” with overall dimensions of ~6.8 nm diameter (at the waist) × ~11.5 nm height (Fig. 2A and B). Around the trimer body, the subunits partially enwrap one another at the base (Fig. 2D). Views of the trimer from above and below each suggest a hexagonal outline (Fig. 2E and F). The upper and lower hexagons superimpose on one another “in phase” as a hexagonal prism shape (Fig. 2G), with a ~60° twist within each individual subunit.

### P4a-3 removal is both necessary and sufficient for P4a-1 trimer formation

All attempts to model a trimer for the full-length P4a precursor failed (data not shown), consistent with which the P4a-3 segment presents a fundamental steric block to subunit mating (Fig. 1B, data not shown). However, trimer models could be readily obtained for both P4a-1 and the P4a-1+2 intermediate (in both pre- and post-rotation conformations about the P4a-1 hinge; Fig. S10). We conclude that the removal of the P4a-3 segment is both necessary and sufficient for P4a-1 trimer formation.

### Trimer molecular model is consistent with cryoEM imaging

Since P4a-1 is considered to lie on the outer surface of the virion core wall (34),we scrutinized published reports for the dimensions of core wall ultrastructural features that might correlate with P4a-1 trimers. Early EM and cryoEM studies of virion cores reported a “palisade” layer of spikes or “pegs” on the outer core wall surface (35 – 38). Measurements of the spike dimensions varied in these early reports, perhaps due to limitations in instrument capabilities, with spike lengths ranging from 100 to 200 Å (36) and diameters of 50–100 Å (39). A very recent cryoEM study (released during the final preparation of this manuscript) of Vaccinia IVs and MVs by Hernandez-Gonzalez et al. (40) measured the palisade layer thickness at 12.5 nm. These measurements agreed closely with the dimensions of our P4a-1 trimer model (Fig. 2). Furthermore, the P4a-1 trimer model’s size and shape were comparable with the numerous scattered features visible in Fig. 8 of reference 38 that could now be identified as probable dissociated P4a-1 trimers (Fig. S11). The above data were consistent with P4a-1 trimer being the primary distinguishable component of the “palisade” spikes.

### Higher-order P4a-1 trimer structure and assembly

We next investigated the higher-order organization of P4a-1 homotrimers, using XLMS data for guidance: Identical peptides cross-linked to one another (homomultimer XL) can have only arisen if the cross-link bridges homomultimer subunits. Our homomultimer XL data set for P4a-1 from intact MV, showed a summed CSM count of 779. While the discrete homotrimer model (above, Fig. 2) satisfied 95.5% of the CSM count for all intra-P4a-1 cross-links (Fig. S12), it could satisfy only 44.7% of the 779 homomultimer CSMs. The remaining 55.3% represented, apparently, undefined higher-order assemblies of P4a-1. Most striking among these was cross-link 366-366, which accounted for nearly half of all P4a-1 homomultimer CSMs, and was detected strongly for all cross-linker types in our data set including those with the shortest cross-linking distances (e.g., PhoX; just 23 Å). Other higher-order homomultimer XL detected in intact MV included 508-508 and 557-557 (CSM count = 66 and 6, respectively).

**Fig 3:**
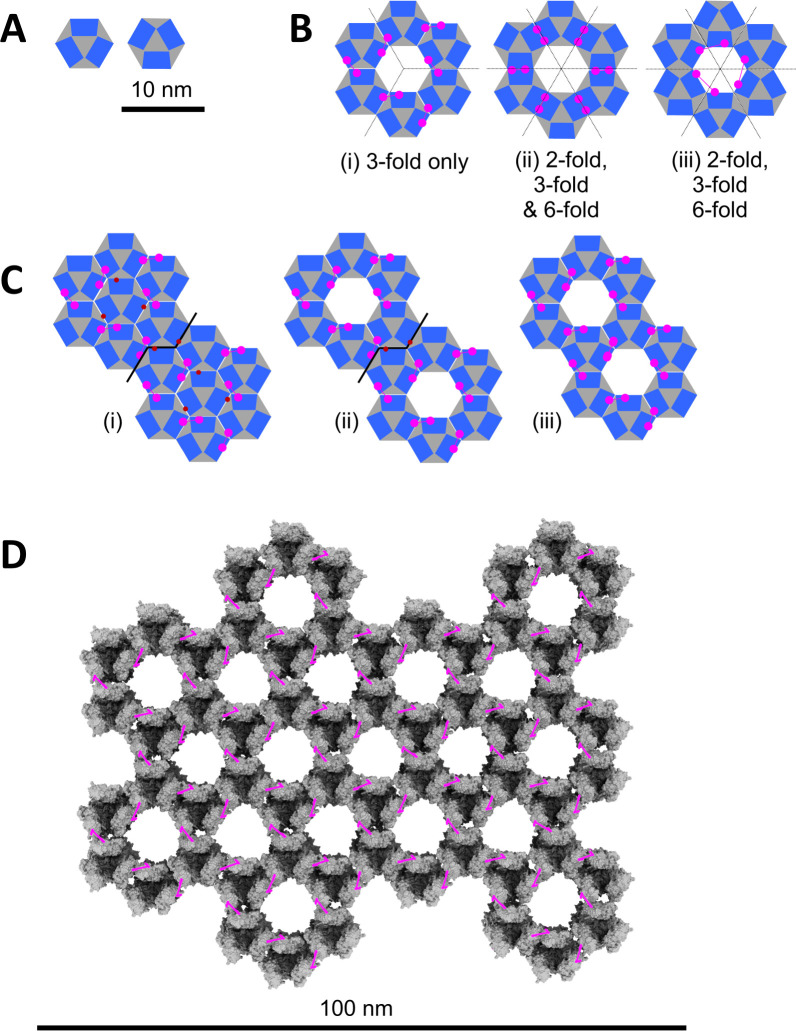
Hexamer-of-trimers models: Genesis, in schematic form. (**A**) The two rotamers of an individual trimer (60° difference). (**B**) Rotationally symmetrical hexamer-of-trimers models based on the trimer’s hexagonal outline (symmetries are indicated). In (i), all trimers are in the same rotational orientation, while in (ii) and (iii), the two forms in panel (**A**) alternate. Internal trimer-trimer 366–366 cross-links are indicated in Magenta (the outward-facing six subunits are unsaturated providing potential hooks to neighboring trimer-hexamers). Cross-link distances favor model (i) over models (ii) and (iii). (**C**) Trimer-heptamer (i) and trimer-hexamer interface schemes (ii, iii) based on the model in panel B(i). Schemes (i) and (ii) do not saturate all interfacial 366–366 XL (unsatisfied cross-linkable sites shown brown), while (iii) does. (**D**) Lattice based on the model in panel (C)(iii). 366–366 cross-links, which reside below the upper surface of the protein when viewed from above (Fig. S14), have been projected over the surface to emphasize their locations. The Euclidian plane tessellation of panel (**D**) has regular p6m symmetry (one-uniform) with periodic (**H and K**) = (3,0). The low confidence C-terminal tails (residues 600–614) were removed for clarity.

The trimer’s hexagonal outline (above; Fig. 2E through G) suggested a higher-order organization comprising some form of hexagonal array. Arranging individual trimers (Fig. 3A) within the simplest conceivable hexagonal array yielded three potential rotationally symmetrical trimer-hexamer models (Fig. 3B). Of these, Fig. 3B(iii) could not satisfy the 366–366 homomultimer cross-link at all. Figure 3B(ii) could satisfy neither 366–366 with cross-linkers PhoX or DSG, nor 508–508 with cross-linkers PhoX, DSA, DSS, or BSPEG5, presenting a fatal limitation for this model. The model of Fig. 3B(i), however, could satisfy 366–366 for all cross-linker types (with a cross-linking distance of just 21.6 Å, Fig. S13) as well as 508–508 and all other higher-order homomultimer XL. This model (Fig. 3B(i)) accounted for 97.2% of all homomultimer XL observed for P4a-1 from MV (either intact or de-enveloped) and was selected as a basis for higher-order modeling (Fig. 3C). Higher-order models comprised a trimer-hexamer whose central hole was filled by a 7th trimer (trimer-heptamer; Fig. 3C(i)) or a tessellation of either abutting or fused trimer-hexamers (Fig. 3C(ii), (iii), respectively). Albeit the trimer-heptamer model could not be fully saturated with 366-366 homomultimer XL (Fig. 3C(i)), this by itself did not render the model invalid. The trimer-heptamer model did, however, lead to an entirely “closed” core wall rather than the more open lattice with trimer-hexamers (Fig. 3C(ii), (iii)), with the former reminiscent of a rigid capsid rather than the flexible, collapsible sac that is more characteristic of the vaccinia core (2, 3).

Between the two trimer-hexamer interface models (abutted or fused, Fig. 3C(ii), (iii), respectively), only the fully fused model (Fig. 3C(iii)), when extended to a larger lattice (Fig. 3D), could fully saturate the lattice with “holes” (Fig. 3D) to combine uniform strength in all directions with maximum flexibility. Also unique to this model: Every trimer fell at the vertex of three fused trimer-hexamers, every trimer-trimer interface separated two holes in the lattice, and three sides of every trimer faced a hole. Moreover, the highest order trimer cluster was simply a trimer-dimer: In contrast to model C(i) and (ii) or mixed forms (not shown), there were no higher-order clusters. Finally, model C(iii) was unique in providing a 366–366 cross-link for every P4a-1 subunit, uniformly saturating the lattice with this XL (Fig. 3D). Model C(iii) was also fully saturated with 508–508 and all other higher-order homomultimer XL. For all the above reasons, model C(iii) was the favored building block for a full core wall lattice (Fig. 3D).

### The higher-order lattice model is consistent with cryoEM imaging

We next attempted to reconcile the trimer-hexamer molecular model with cryoEM images of the intact virion core wall. Dubochet (38), Cyrklaff (41), and Hernandez-Gonzalez (40), reported patches of hexagonal features on the surface of the core wall, identified as the “palisade” coated with pegs (41), all of which fit and scaled to our P4a-1 lattice (Fig. S14). Pegs visible in relief around the edges of the virion core in cryoEM images seemed to have a periodicity of ~8.5 nm, comparable to that of both the hexagonally arranged features in the same cryoEM images (38, 41) and individual P4a-1 trimers in our lattice molecular model (Fig. S14). At a specific imaging orientation (Fig. 3D), trimer electron density may be expected to sum through sequential rows of trimers to yield the “palisade” effect.

**Fig 4:**
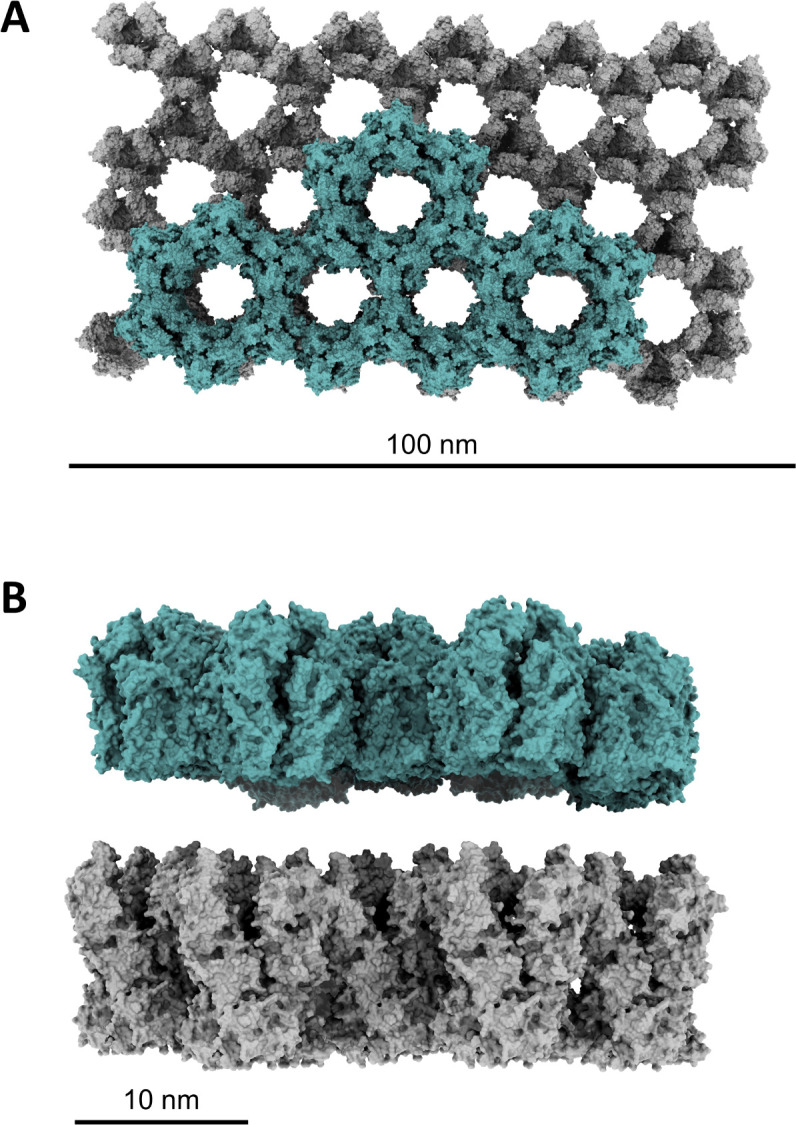
Modeled lattice of P4a-1 is co-dimensional with the D13 external scaffold (42). Gray: P4a-1. Turquoise: D13. View from above (**A**) and side in higher zoom (**B**). In panel (**B**), P4a-1 and D13 lattices are separated by an arbitrary distance of 5 nm.

Published cryoEM images show hexagonal features only discontinuously across the core (38, 41). This could arise from overlaying features (such as bent or distorted pegs), the technicalities of cryosectioning, or because the P4a-1 lattice is discontinuous. To establish whether the lattice can be reasonably considered to cover the entire surface of the core, we derived the anticipated experimental mass of P4a-1 in the virion based on known virion mass, the proportion of total mass comprising protein, and proportion of total virion protein comprising P4a-1 (Supp. Doc. 1). This calculation indicated that 12.4% of the overall virion mass, or 404 MDa, comprised P4a-1. In an independent calculation for the theoretical mass of P4a-1 in the virion if it were enclosed entirely by our lattice, we calculated the surface area of a virion core (taking the mean core size from a number of published images) and the total number and then mass of P4a-1 molecules that could coat it fully according to the lattice of Fig. 3D. This totaled 6,064 molecules or 430 MDa of P4a-1 (Supp. Doc1). From the similarity of the two values (404 and 430 mDa), we conclude that the modeled P4a-1 lattice likely encloses the entire surface of the virion core, albeit with, perhaps, localized regions of disruption. In this regard, for example, “pore-like” features on the surface of the core wall (34, 40, 41) may contribute to the disruption of the P4a-1 lattice.

### Correspondence of P4a trimer lattice to the external scaffold

Vaccinia morphogenesis initiates with the insertion of virion envelope proteins A17 and A14 into the ER membrane. This membrane is subsequently fractured, allowing A17 to associate with trimers of Vaccinia external scaffold protein D13, resulting in spherical IVs coated with the D13 external scaffold (1, 43). Deep-etch EM has shown that the external scaffold forms a hexagon-like honeycomb lattice across the entire surface of IV, with pentameric and heptameric defects (43). The known experimental structure of the external scaffold also shows D13 trimers forming a hexagonal trimer-hexamer lattice (42). During maturation from IV to MV, the A17 N-terminus is cleaved by Vaccinia protease I7, leading to D13 scaffold release. Either simultaneously or soon thereafter, the A17 C-terminus and core structural proteins (including P4a) are processed, resulting in condensation of the core wall and palisade layer (20, 24 – 29). Hexagonal features of the palisade layer, visualized by cryoEM (40, 41) are reminiscent of the honeycomb D13 lattice. Our P4a-1 trimer-hexamer lattice model was, dimensionally, entirely coincident with the atomic structure of the D13 lattice (Fig. 4). In some manner, the external scaffold, through its interaction with A17, may template condensation of the P4a-1 lattice during virion morphogenesis, for subsequent core wall assembly.

## DISCUSSION

There are currently atomic-level structures for all or parts of 34 of the ~75 packaged Vaccinia gene products. However, with perhaps one exception, namely the packaged multisubunit Vaccinia RNA polymerase (44), how these proteins condense into higher-order assemblies has eluded analysis. Here, we have investigated Vaccinia major structural protein P4a, which accounts for 12.4% of the total virion mass. P4a precursor is transported to IVs during assembly where it is proteolytically processed at two AG| sites (19). P4a-1 and P4a-3 are packaged in MVs, while P4a-2 is discarded (19). The biological and functional significance of the two processing events has not been previously elucidated. Moreover, how P4a-1 forms the core wall and/or palisade layer associated with the core wall has remained unresolved. Here, we have combined deep-learning protein structure prediction with a deep XLMS data set comprising ~135,000 cross-link spectral matches via 10 distinct chemical cross-linkers to yield ~22,000 unique-mass cross-linked peptide pairs. The predicted structure for P4a-1 monomer was consistent with all intra-protein cross-links (Fig. 1; Fig. S3). Homomultimer models were optimal at the trimer level (Fig. 2; Fig. S8 and S13) and the resulting trimer model was consistent with published low-resolution cryoEM images (Fig. S11) but did not accommodate all cross-links of the inter-subunit type. Building a hexamer of trimers of a specific topology, however, allowed essentially all inter-subunit cross-links to be satisfied and they remained satisfied upon building the trimer-hexamer to a full core wall lattice (Fig. 3; Fig. S13 and S14).

**Fig 5:**
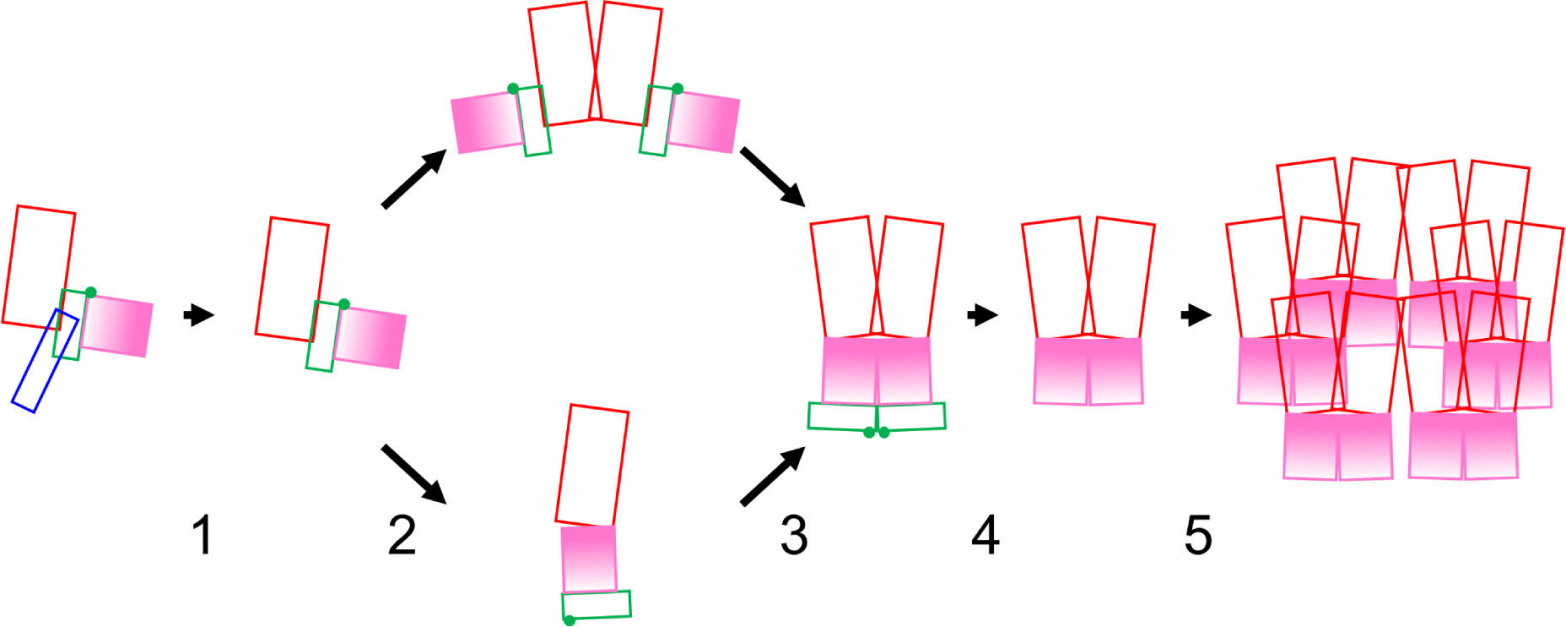
Suggested five-step pathway of P4a processing, conformational change and assembly. Coloration (as in Fig. 1A): P4a-1 domain I red, P4a-1 domain II pink, P4a-2 green, and P4a-3 blue. P4a-1 domain II is shown with gradient fill to emphasize its conformational inversion at steps 2/3. Green spot: P4a-1|two processing site. In step 1, P4a-3 removal permits both conformational inversion and trimerization of the P4a-1+2 intermediate (steps 2 and 3). The order of steps 2 and 3 is undefined hence the upper and lower loops of the pathway. In step 4, P4a-1|two processing permits higher-order P4a trimer assembly (step 5). For simplicity, only two of the three trimer subunits are shown in multimer schematics. For details see text.

We suggest a five-step ordered/concerted assembly pathway for P4a-1 (Fig. 5; Movie S2). In the initial P4a precursor-monomer conformation (prior to step 1), the P4a-3 segment presents a steric block to trimerization and the P4a-1 trimerization interface is conformationally split. P4a-3 detachment by AG| processing initiates the pathway (step 1), relieving the block to trimerization, while also destabilizing the precursor conformation of the P4a-1 segment leading to the massive conformational rotation of P4a-1 domain II (Fig. 5, steps 2 and 3). We cannot distinguish the temporal order of trimerization and conformational inversion without evidence that either is dependent on the other: Models for both P4a-1+2 monomer and trimer showed both pre- and post-rotation conformations (Fig. 5, step2-lower or step3-upper, Fig. S10 and S2), and both conformations were able to trimerize (Fig. 5, step2-upper or step3-lower, Fig. S10). Perhaps the two events occur simultaneously. After conformational inversion, P4a-1 domains I and II pack together (Fig. 1B) and become conformationally locked by disulfide bond formation between Cys31 and Cys569 (Fig. S7). In addition, P4a-1 domain I and II helices now pack together allowing new interactions between them (Fig. 1B). The P4a-2 segment is now isolated at the end of the molecule (Fig. 5, steps 2/3). Since a trimer could be modeled for not only fully processed P4a-1 but also the P4a-1+2 intermediate (Fig. 5, before/after step 4) albeit with a lower confidence PAE, excision of the P4a-2 segment is not an absolute prerequisite for P4a-1 trimerization. P4a-2 excision is, however, a prerequisite for higher-order trimer assembly: after conformational inversion in the P4a1+2 trimer (Fig. 5, step3-upper) removes one steric block to higher-order assembly (Fig. 5, step 3), a steric block remains after step three due to presence of the P4a-2 segment on the outside surfaces of P4a-1+2 trimer subunits coincident with sites of higher-order assembly. In step 4 (Fig. 5), release of the P4a-2 segment via processing at the 1|2 AG| site allows higher-order assembly into a trimer-hexamer lattice (Fig. 5, step 5). The overall pathway (Fig. 5) is animated in Movie S2.

In studies with wild-type MV in the infected cell (19), anti-P4a-2 antibody could detect the P4a-1+2 intermediate but not P4a-2+3, suggesting that processing to remove P4a-3 precedes processing to split P4a-2 from P4a-1. Our pathway reflects this and now can be rationalized as the deferral of higher-order assembly until after trimerization has occurred. A model in which this order of processing events is regulated sterically (steric occlusion of the 1|two processing site until after 2|three processing and the resulting conformational inversion) is not viable since both P4a processing sites appear to be solvent exposed in all conformers and intermediates (Fig. 1). We therefore suggest an alternative model in which the three 1|two processing sites of a trimer must be brought into spatial proximity (at a point in space beneath the trimer’s center; Fig. 5, step 3) for 1|two processing to occur. This would be the case if, for example, the processing protease is spatially restricted instead of freely diffusible, and would then defer P4a-2 removal (the last block to higher-order trimer assembly) to the final portion of the pathway.

## MATERIALS AND METHODS

### Protein structure prediction, *de novo*

Monomer and multimer structure predictions for vaccinia protein P4a (precursor and processing products) were run on local installations of AlphaFold2 and AlphaFold-multimer, using a non-docker setup (https://github.com/kalininalab/alphafold_non_docker). Models shown here represent the top-ranked prediction for each protein or protein complex. Side chains were present in models but not rendered except where such rendering was chosen. For generating 20 models from AF2 (e.g., Fig. S3) where it would otherwise produce only produce five models AlphaFold2 was run four times and the resulting models were pooled, or AlphaFold-multimer was run for the target protein. For each proteoform (P4a precursor, P4a-1+2 intermediate, mature P4a-1, and P4a-1 trimer), the top-ranked structure was compared individually against the other 19 models using ChimeraX MatchMaker. The RMSD (Å) between all-carbon atom pairs, per comparison, was reported as a data point, with 19 total data points for each model set.

Structure predictions through RoseTTAFold were run on the Robetta server (https://robetta.bakerlab.org/) using standard parameters.

### Scoring of structural models

Average pLDDT scores for structures predicted with AlphaFold2 were obtained by averaging pLDDT values for all-carbon atoms (representing all atoms) from the top predicted model for each structure (in many cases, some top models were either identically or almost identically scored).

### Visualization

Reported structures were visualized and measured, and images were generated using UCSF ChimeraX (45, 46). PAE plots of AlphaFold-multimer structures were visualized by the “AlphaFold Error Plot” tool provided by ChimeraX.

### Cross-linking mass spectrometry

Virion proteins were cross-linked, prepared for XL-MS, and analyzed as described in greater detail in reference (6). Briefly, bis(succinimidyl) penta(ethylene glycol) (BSPEG5), bis(succinimidyl) nona(ethylene glycol) (BSPEG9), adipic acid dihydrazide (ADH), 1-ethyl-3-(3-dimethylaminopropyl)carbodiimide hydrochloride (EDC), and n-hydroxysuccinimide (NHS) were purchased from ThermoFisher, Inc. Isotopically coded disuccinimidyl suberate (DSS), bis(sulfosuccinimidyl)suberate (BS3), disuccinimidyl glutarate (DSG), and disuccinimidyl adipate (DSA) were purchased from Creative Molecules. 3,5-bis(((2,5-dioxopyrrolidin-1-yl)oxy) carbonyl)phenyl)phosphonic acid (PhoX) was provided by the Scheltema lab and later purchased from Bruker and prepared as described in reference (45). Vaccinia virus was grown in HeLa S3 cells (ATCC CCL-2.2) and purified over a 36% sucrose cushion, followed by two 24–40% sucrose gradients. The harvested virus was washed with 100 mM triethylammonium bicarbonate buffer (TEAB) pH 8.5 and cross-linked as intact virions or after brief uncoating treatment (0.05% NP40, 40 mM TCEP, 100 mM TEAB, pH 8.5). Appropriate XL concentrations were determined by SDS-PAGE. XL reactions were quenched with ammonium bicarbonate or removed by spin desalting into ammonium bicarbonate.

Cross-linked virus samples were disaggregated by various methods, including an in-lab modified FASP protocol and urea denaturation. Cleavage of solubilized proteins into peptides employed various reagents/reagent combinations: Trypsin, ArgC, GluC, AspN, LysC, Trypsin+LysC, Trypsin+GluC, Trypsin+AspN, ArgC+AspN, ArgC+GluC, AspN+GluC, or CNBr+Trypsin. All cleaved samples were acidified with formic acid (FA) to 2% FA final concentration and desalted by C18/SCX as described (47). Peptides were eluted with 5% NH4OH, 80% CH2CN, 0.1% FA (Buffer X) or with a six-step ammonium acetate gradient in 20% CH3CH, 0.5% FA, followed by a final elution with Buffer X. Samples were dried under vacuum and reconstituted in 0.1% FA in water for MS. The nanoLC-MS/MS method used is described in reference (6).

Instrument raw files were converted to mgf or mzML using MSConvert by ProteoWizard. Cross-links were identified by the programs described in reference (6) with the addition of pLink2, Kojak2, MetaMorpheus, and XlinkX. The in-house code described in reference (6), with substantial upgrades including for large data sets, was used to consolidate and assess XLMS data and calculate CSM counts. The latter represents total CSMs for a particular cross-link or set of cross-links.

Cross-link networks were rendered using CrosslinkViewer (48). Solvent-accessible surface distances between cross-linked residues were calculated using Topolink (49). Models of cross-linked pairs with through-space (Euclidean) distances indicated were rendered using ChimeraX.

### Feature size measurement (EM)

Spike lengths and diameters were measured using the “tape” feature of ChimeraX: images from publications by these groups were saved as TIFF files, opened in ChimeraX, and spike lengths and diameters were measured with the ChimeraX Tape feature. The provided scale bars were used to convert the ChimeraX measurement values to Å.

## Data Availability

All structural model coordinates and FASTA files are available on DRYAD. All additional materials are available from the authors by reasonable request.

## AFTER EPUB

*[This article was published on 30 August 2023 with typographical errors in Results, Fig. 1 and its legend, and the supplemental material. The errors were corrected in the current version, posted on 12 September 2023.]*

## SUPPLEMENTAL MATERIAL

Document S1 mbio.01135-23-s0001.docx Calculation of P4a-1 coverage of virion core wall surface.

Supplemental figures mbio.01135-23-s0002.docx Figures S1 through S14.

Movie S1 mbio.01135-23-s0003.mp4 Proposed conformation change in P4a during AG| processing to the three known products.

Movie S2 mbio.01135-23-s0004.mp4 Proposed overall pathway of P4a processing, conformation change, and assembly.

ASM does not own the copyrights to Supplemental Material that may be linked to, or accessed through, an article. The authors have granted ASM a non-exclusive, world-wide license to publish the Supplemental Material files. Please contact the corresponding author directly for reuse.
