# Supplementary material for: Combination of deep XLMS with deep learning reveals an ordered rearrangement and assembly of a major protein component of the vaccinia virion
Source: mBio. 2023 Aug 30;14(5):e01135-23. doi: 10.1128/mbio.01135-23 (PMC10653903; doi:10.1128/mbio.01135-23)
Supplement: Document S1 — Calculation of P4a-1 coverage of virion core wall surface. [file mbio.01135-23-s0001.docx]

**Supplementary Document 1**

Core surface area

Dumbell core as rectangle: 245 x 62 x 135 nm (mean values of measurements from several publications)

Two faces 245 x 62 = 30,380

Two faces 62 x 135 = 16,740

Two faces 245 x 135 = 66,150

Total: 113,270 nm^2^ = ~0.113 mm^2^

Virion molecular composition

Assuming 5 x 10^6^ lipid molecules per mm^2^ of typical biological membrane:

https://www.ncbi.nlm.nih.gov/books/NBK26871/

Vaccinia envelope has 0.113 x 5 x 10^6^

= **5.65 x 10^5^ lipid molecules**

Lipid composition (plasma membrane):

https://www.britannica.com/science/lipid/Lipids-in-biological-membranes

Average lipid Mr based on plasma membrane composition:

|  | **% composition** | **Mr** | **mole%** |
| --- | --- | --- | --- |
| Phosphatidyl choline | 31% | 314.25 | 97.34 |
| Cholesterol | 28% | 386.7 | 108.28 |
| Sphingomyelin | 16% | 493.6 | 79 |
| Phosphatidylethanolamine | 14.3% | 299.21 | 42.76 |
| **TOTAL:** | **89%** |  | **327.38** |

327.38 / 0.89 (since above accounts for 89% of total lipid): **367.8 (average Mr)**

5.65 x 10^5^ x 367.8 = **207.8 MDa** of lipid in virion

Genome MW = 190,000 x 333 = **126 mDa** DNA in virion.

**Virion dry protein mass calculation**

Virion dry molecular mass = 3.26 GDa

Molecular mass of protein = virion dry mass – (lipid + DNA) = 3,260 – (207.8 + 126) Mda = **2,926 MDa protein**

13.8% (by mass) of total protein = P4a-1 (*1*)

0.138 x 2,926 = **404 MDa** of virion Mr = P4a-1

Parenthetically:

Protein content of virion from above calculation = 2,926 / 3,260 = 89.75% protein. Compared to:

The composition of the Vaccinia particle is 90% protein, 5% lipid and 3.2% DNA (*2*).

**Molecular counting calculation, assuming the core wall lattice model entirely encloses the core:**

Area of 10-hole lattice segment in molecular model

Lattice (10 holes) falls within a 69.5 x 24.2 nm = 1,681 sq. nm box.

Total #holes in core wall

(Surface area of core / surface area of 10 holes) x 10 = (113,270/1681) x10 = 673 holes enclosing the core

Each hole represents addition of 3 unique trimers to lattice = 9 P4a-1 molecules

So, 9 x 673 = 6,064 P4a-1 molecules, if lattice entirely encloses the core.

Given Mr of each P4a-1 = 71 kDa, then mass of 6,064 P4a-1 molecules = **430 MDa**

Parenthetically: 430 MDa = 13.2% of total virion dry mass; 6,064 P4a-1 molecules = 62 million atoms.

The similarity of the two numbers (404 vs. 430 MDa) suggests P4a-1 forms a near-complete mesh around the core (as one might expect) with localized areas of disruption.

**References:**

1. Sarov, I. and Joklik, W.K., Studies on the nature and location of the capsid polypeptides of vaccinia virions. Virology, 1972. 50(2): p. 579-92.
2. B. Moss, in *Fields Virology*. (Wolters Kluwer | Lippincott, Williams & Wilkins, Philadelphia, 2013), pp. 2129-2159.
