# Supplementary material for: Combination of deep XLMS with deep learning reveals an ordered rearrangement and assembly of a major protein component of the vaccinia virion
Source: mBio. 2023 Aug 30;14(5):e01135-23. doi: 10.1128/mbio.01135-23 (PMC10653903; doi:10.1128/mbio.01135-23)
Supplement: Supplemental figures — Figures S1 through S14. [file mbio.01135-23-s0002.docx]

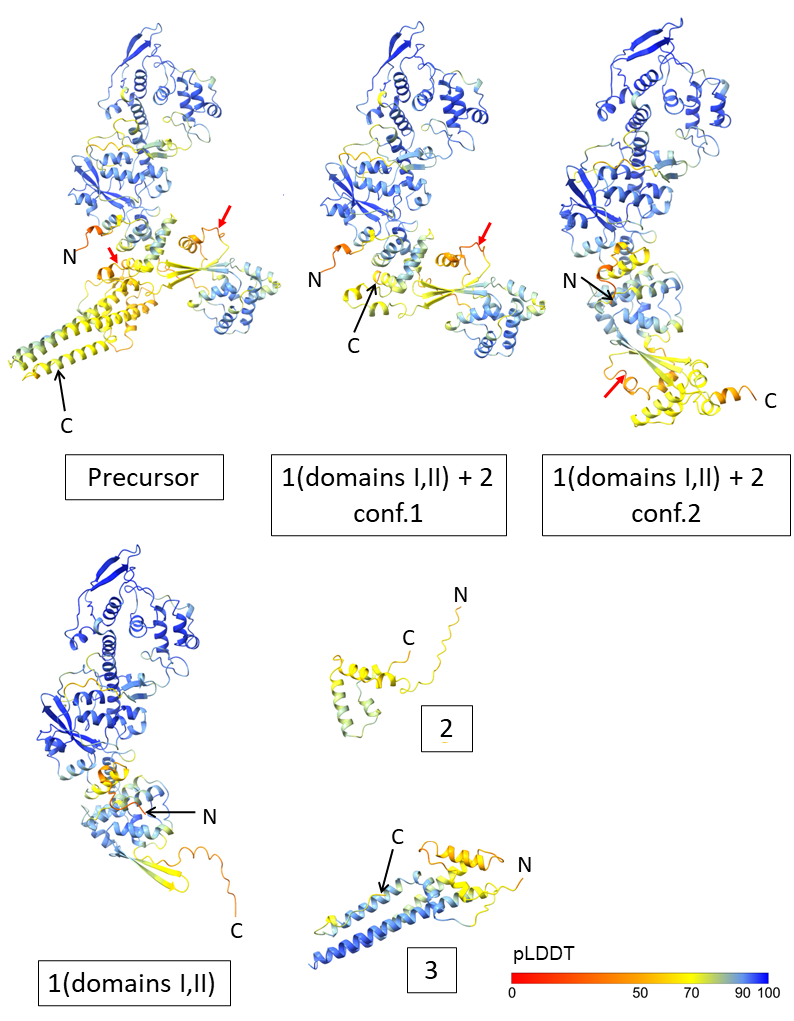


**Fig. S1**.

**Structural models for P4a precursor and processing products and intermediates colored by pLDDT and labeled according to Fig. 1.** Red arrows point to AG| processing sites. Average pLDDT values for P4a-precursor, P4a-1 and P4a-3 were 78.0, 86.5 and 78.2 respectively, with a value of 83.3 for the P4a-1 segment of P4a-precursor. The P4a-1 segment of the cleavage intermediate P4a-1+2 (both forms) had an average pLDDT of 83.5. High confidence extended throughout the structures.


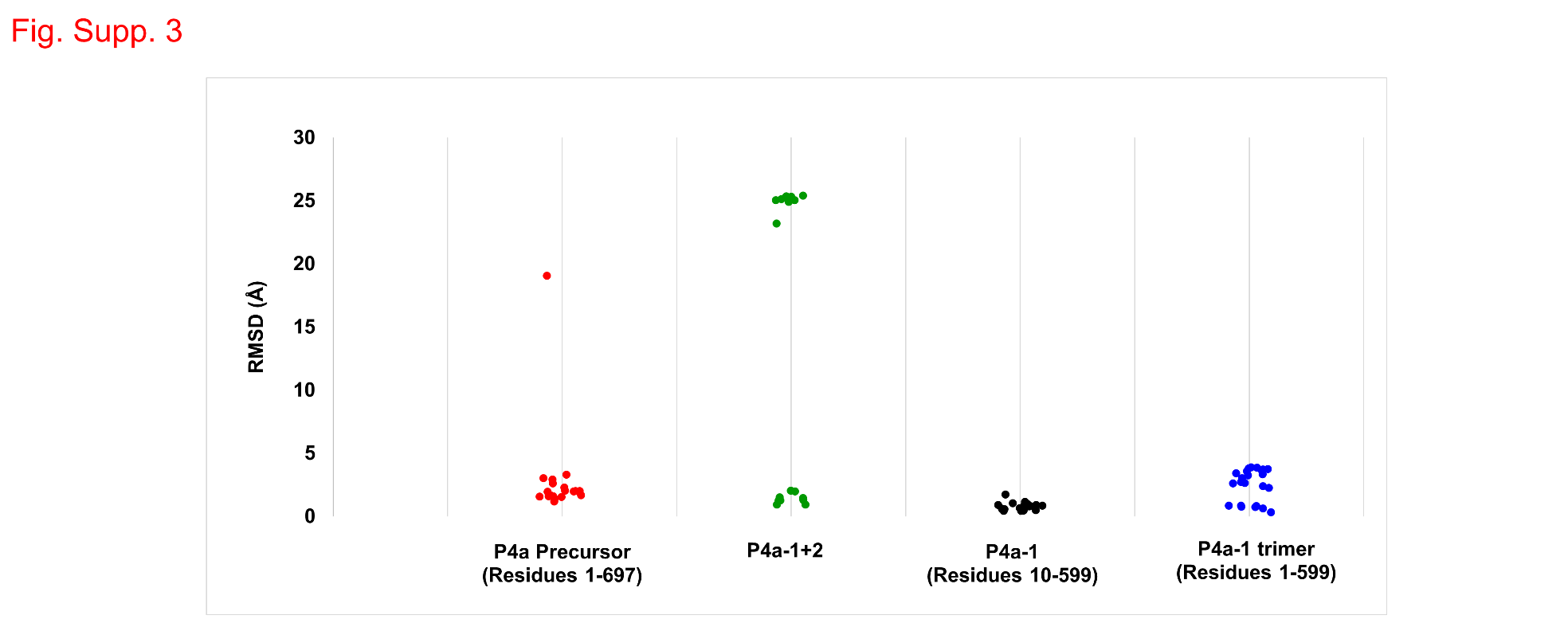


**Fig. S2**.

**Four modeling exercises via AlphaFold-2 or AlphaFold-multimer from each of which the top 20 structural models were considered.** Each data point (Y) represents the RMSD (Å) across all ⍺-carbon atom pairs between the top ranked model and one of the remaining 19 models within the same modeling exercise. Models for P4a-1+2 fell into either of two conformers, namely that for precursor (Fig. 1B, “1(domains I,II) + 2, conf.1”; lower cluster, < 5 Angstroms RMSD, 10 models) or that for mature P4a-1 (Fig. 1B. “1(domains I,II) + 2, conf.2”; upper cluster, > 20 Angstrom RMSD, 10 models). Colors were chosen randomly.


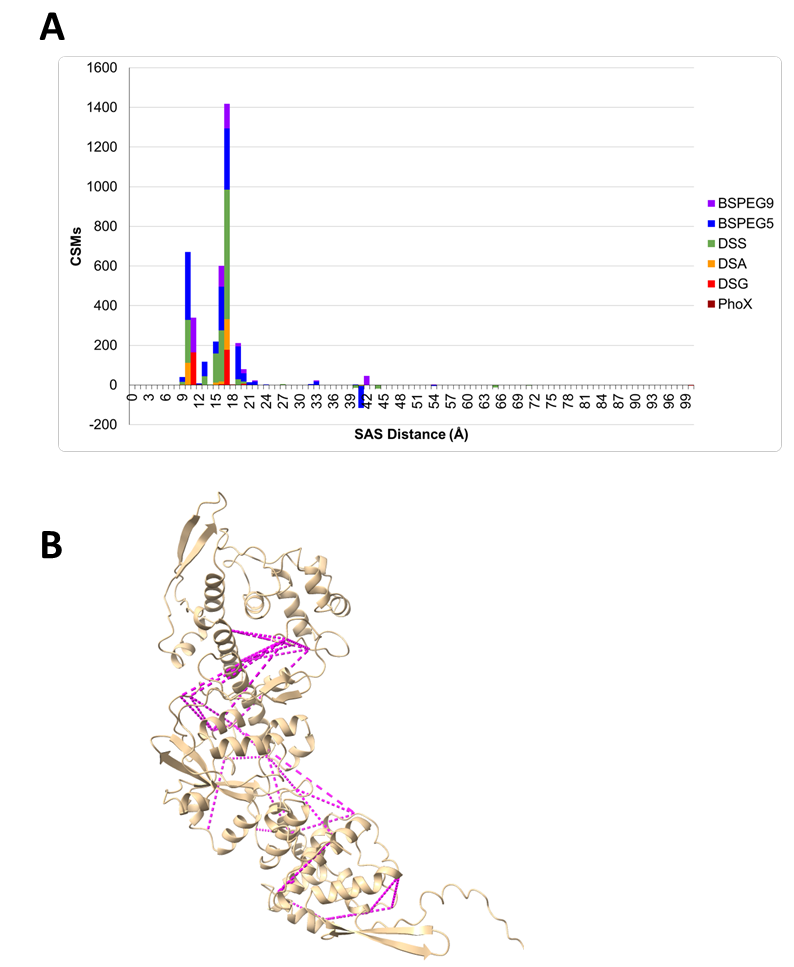


**Fig. S3**.

**P4a-1-localized intramolecular crosslinks in intact MV vs. the AlphaFold2 model of the processed P4a-1 segment of P4a.** 95.5% of the total CSM count from intra-protein crosslinks from intact MV were distance non-violators. (**A**) Solvent-accessible surface (SAS) crosslinking distance vs. CSMs. Colors represent crosslinkers given in the graph legend. Positive and negative values represent, respectively, non-violators and violators for the individual spans of individual crosslinkers. (**B**) Non-violating crosslinks (magenta) mapped on model. In contrast to AlphaFold2, RosettaFold failed to generate a model that could satisfy XLMS data (Figs. S5, S6).


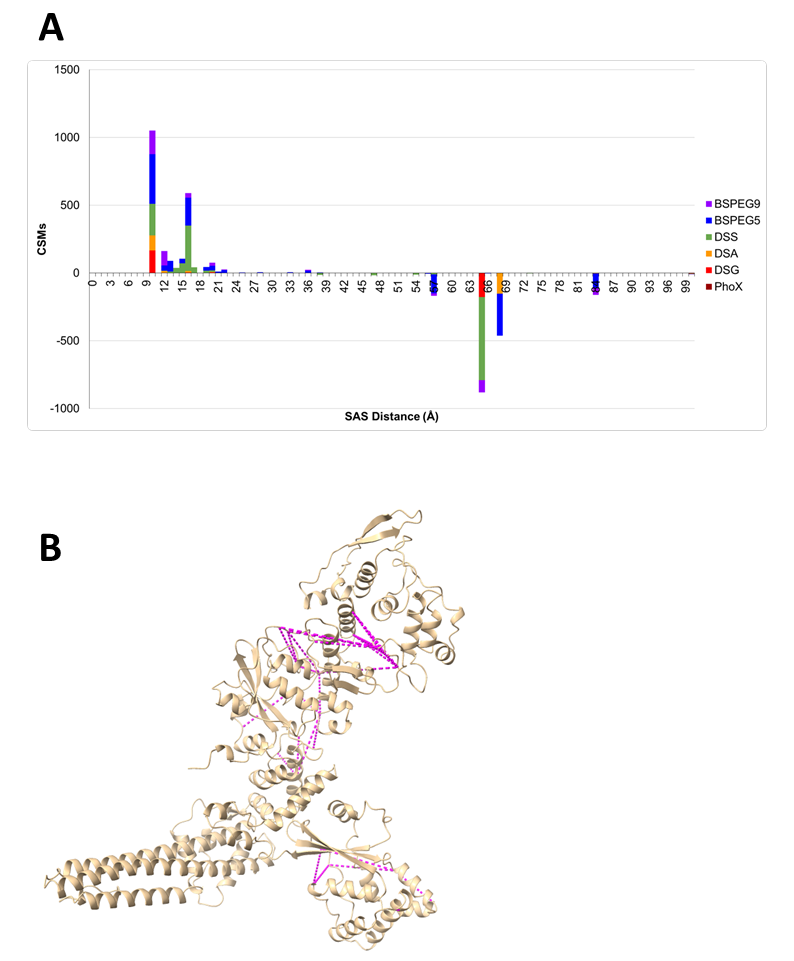


**Fig. S4**.

**P4a-1-localized intramolecular crosslinks in intact MV vs. the AlphaFold2 model of P4a precursor.** Just 56.5% of the total CSM count from intra-protein crosslinks from intact MV were distance non-violators. Details as in Fig. S3.


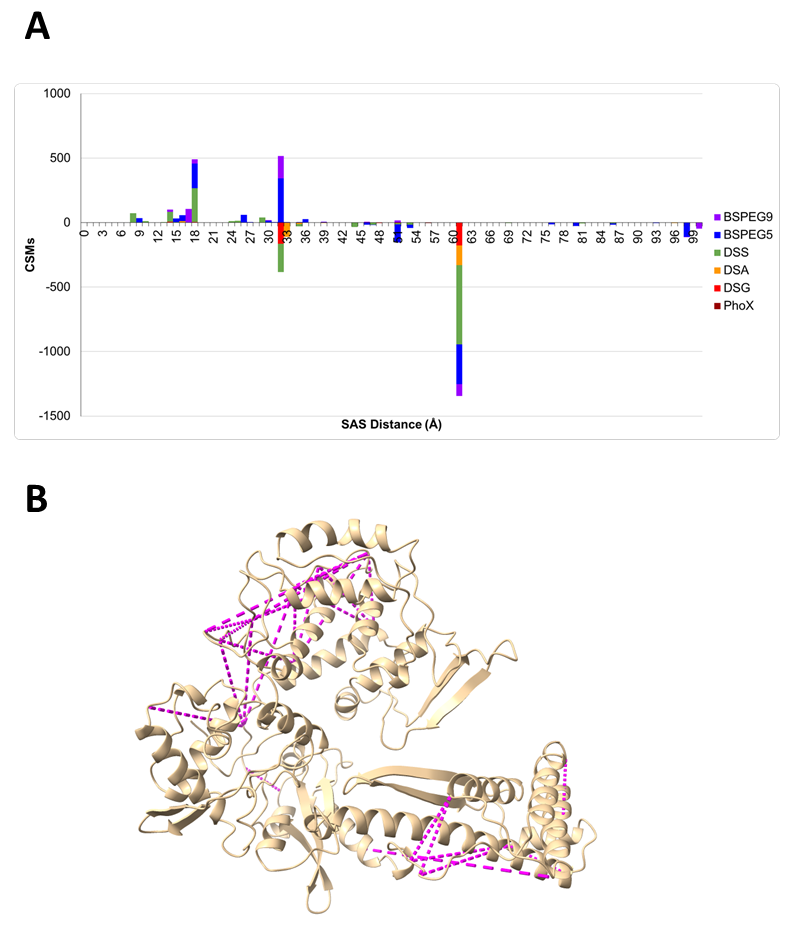


**Fig. S5**.

**P4a-1 localized intramolecular crosslinks in intact MV vs. the RosettaFold model of the processed P4a-1 segment of P4a.** Just 41% of the total CSM count from intra-protein crosslinks from intact MV were distance non-violators. Details as in Fig. S3.


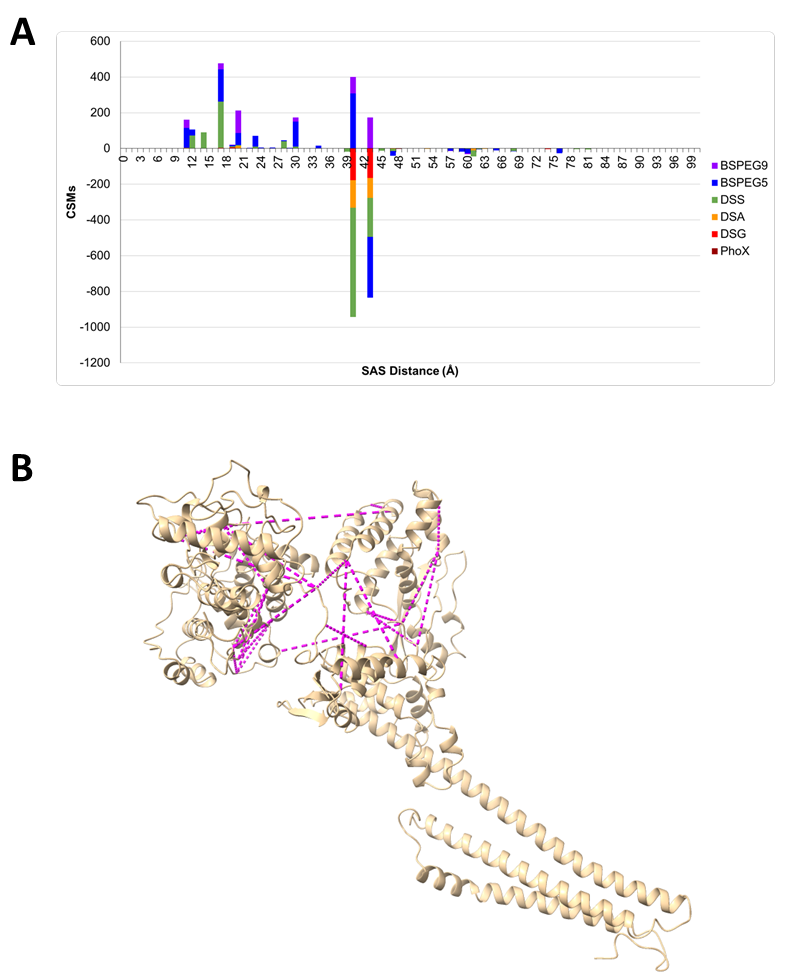


**Fig. S6**.

**P4a-1 localized intra-protein crosslinks in intact MV vs. the RosettaFold model of P4a precursor.** 49% of the total CSM count from intra-protein crosslinks from intact MV were distance non-violators. Details as in Fig. S3.


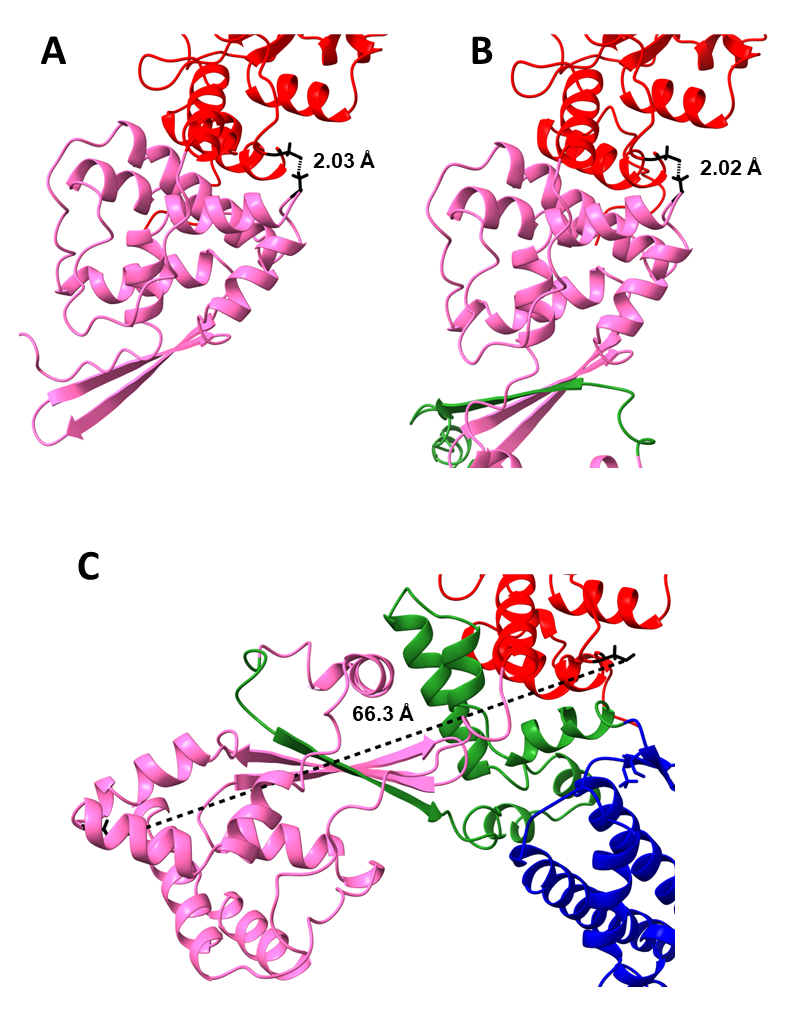


**Fig. S7**.

**Disulfide locking:** Distance between the sulfur atoms of Cys31 and Cys569 in P4a models. (**A**) P4a-1. (**B**) P4A-1+2 intermediate (conf.2). (**C**) P4a precursor. Cys31-Cys569 distances are 2.03 Å, 2.02 Å and 66.3 Å, respectively.


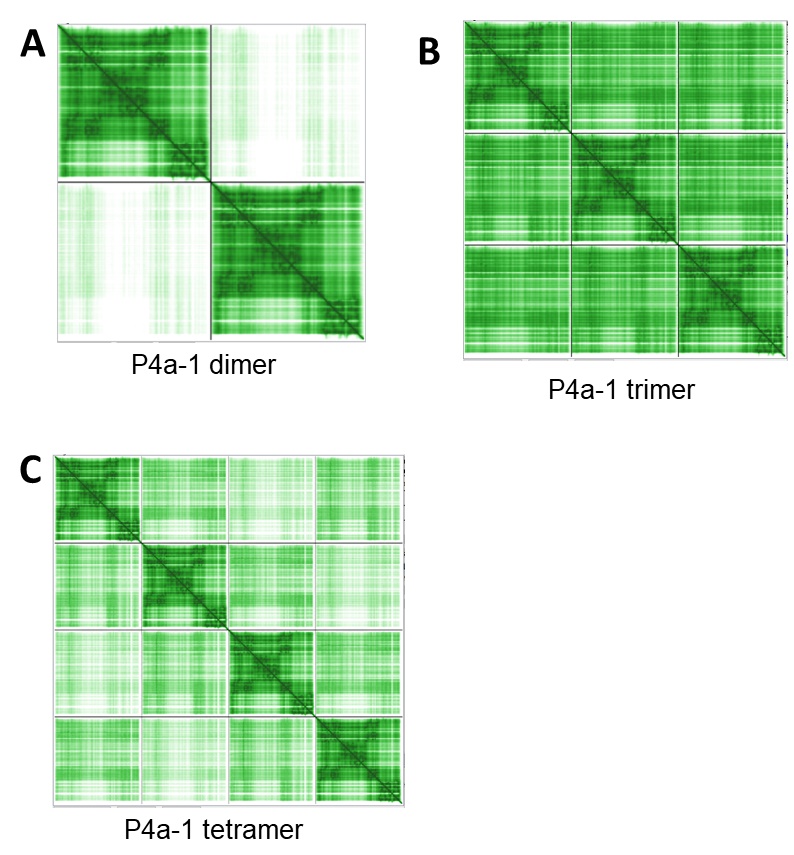


**Fig. S8**.

**Multimer predictions of P4a-1 by AlphaFold-multimer: PAE plots.** (**A**) - (**C**) P4a-1 dimer, trimer, and tetramer models respectively. A sharp green/white delineation at subunit boundaries indicates low confidence in subunit condensation and placement. A smooth green/green boundary indicates high confidence. Among the three models, only the trimer model showed high confidence, comparable to the confidence of the monomer fold.

**
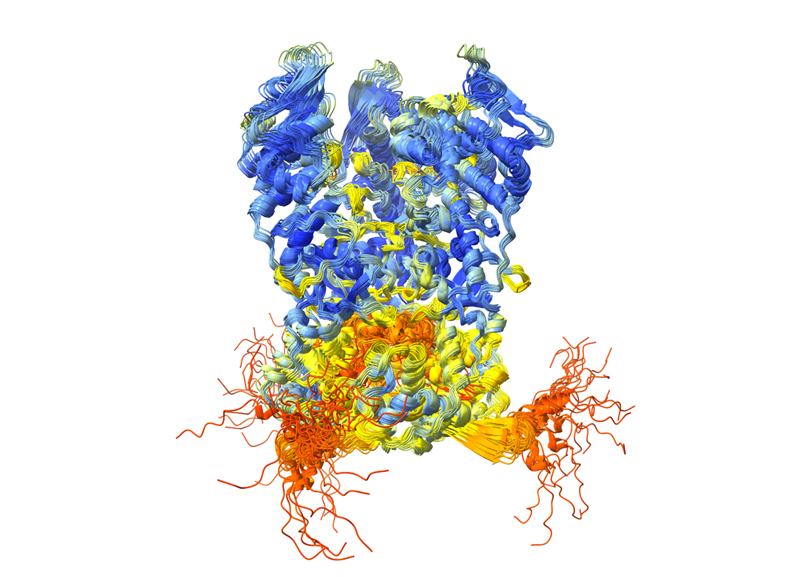
**

**Fig. S9**.

**Superposition of all 25 AlphaFold-multimer models for P4a-1 trimer, colored by pLDDT.** Models showed a high degree of convergence upon a single optimal solution (with exception of the 15 aa flexible C-terminal tail). No inter-subunit disulfide bonds were apparent in the trimer models. RMSD for the top 20 of these models (minus the C-terminal tail) is shown in Fig. S2.

**
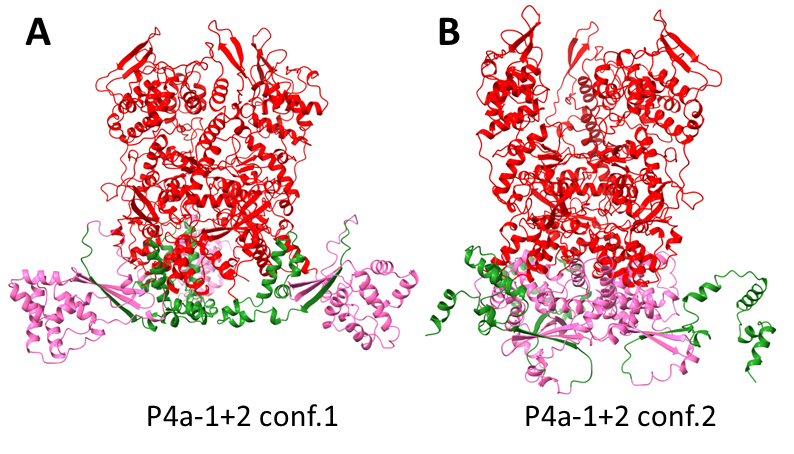
**

**Fig. S10**.

**Trimer models for the P4a-1+2 intermediate predicted by AlphaFold-multimer fell in two conformations about the hinge joining P4a-1 domains I and II.** (**A**), (**B**) The two conformations: Pre- and post-rotation about the hinge, respectively. Domain coloration follows Fig. 1 (Red: P4a-1 domain I (aa 1 - 451); Pink: P4a-1 domain II (aa 452-614); Green: P4a-2 (aa 615 - 697)).

**
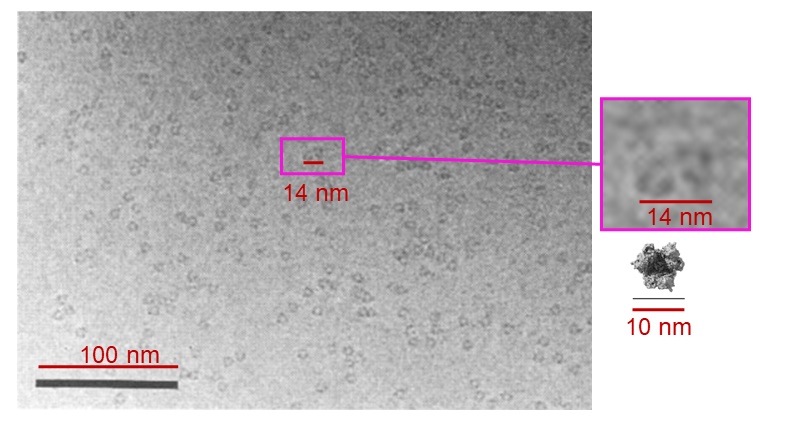
**

**Fig. S11**.

**Comparison of the P4a-1 AlphaFold-multimer molecular model with prior cryoEM images.** (A) Left: A series of identical features scattered on cryoEM grid upon virion uncoating from Dubochet (left) (*38*), enlarged. Upper right: Zoom of a pair of features with proportionately enlarged scale bar. Lower right: Single trimer model (AlphaFold-multimer) to same scale.

**
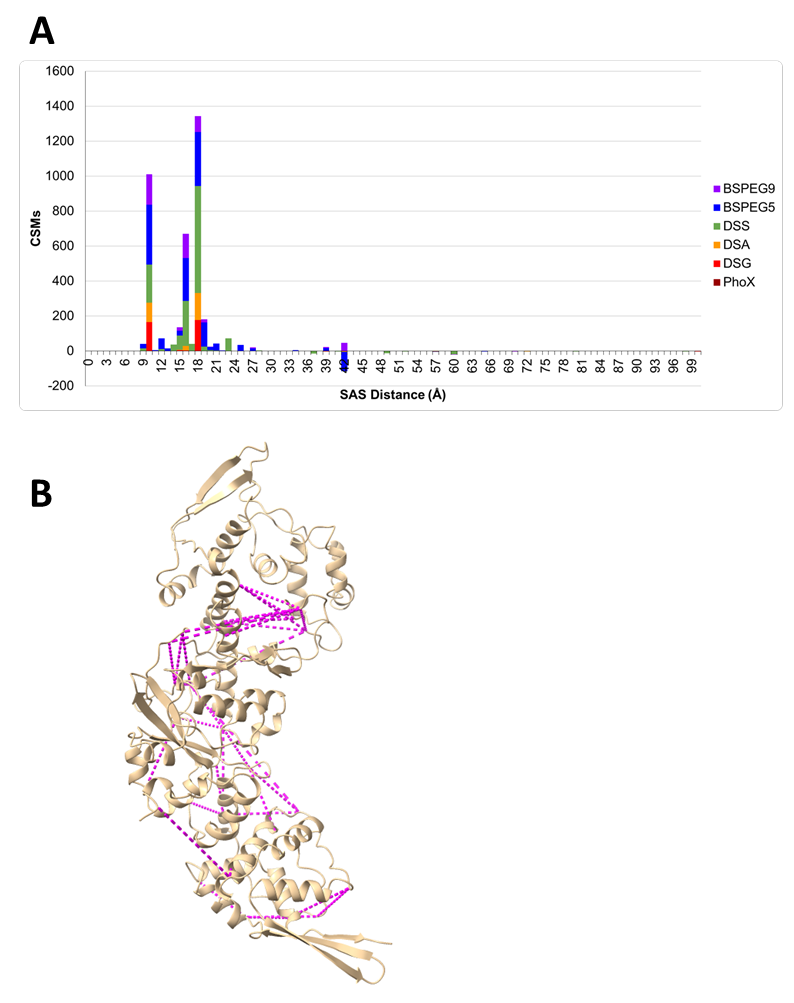
**

**Fig. S12**.

**P4a-1-localized intramolecular crosslinks in MV vs. one subunit from the AlphaFold-multimer model of P4a-1 trimer.** Details as in Fig. S3.

**
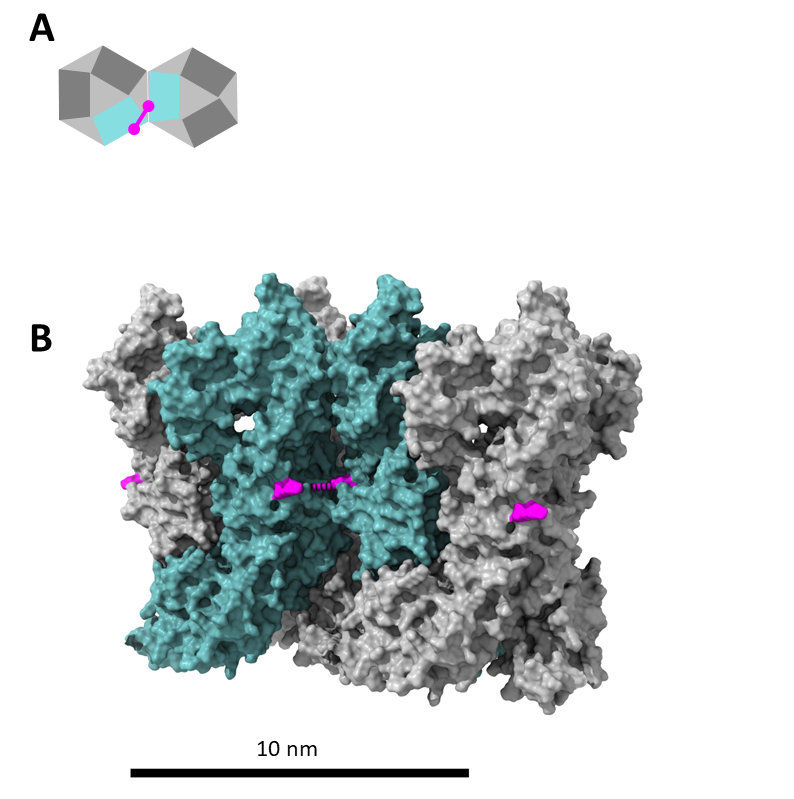
**

**Fig. S13**.

**P4a-1 trimer-dimers.** (**A**) Two P4a-1 trimers (viewed from above in schematic form) brought into a side-by-side alignment that can rationalize the 366-366 homomultimer crosslink; crosslinked subunits shown in blue. (**B**) Crosslinked dimer-of-trimers (side view of panel (A), with residues 600-614 excluded from view) – Blue represents crosslinked subunits. Residues 366 from both trimers colored magenta; the pair within crosslinking range is connected as a crosslink (also magenta). Solvent accessible surface distance is 21.6 Å for this pair (median 24.6 Å).

**
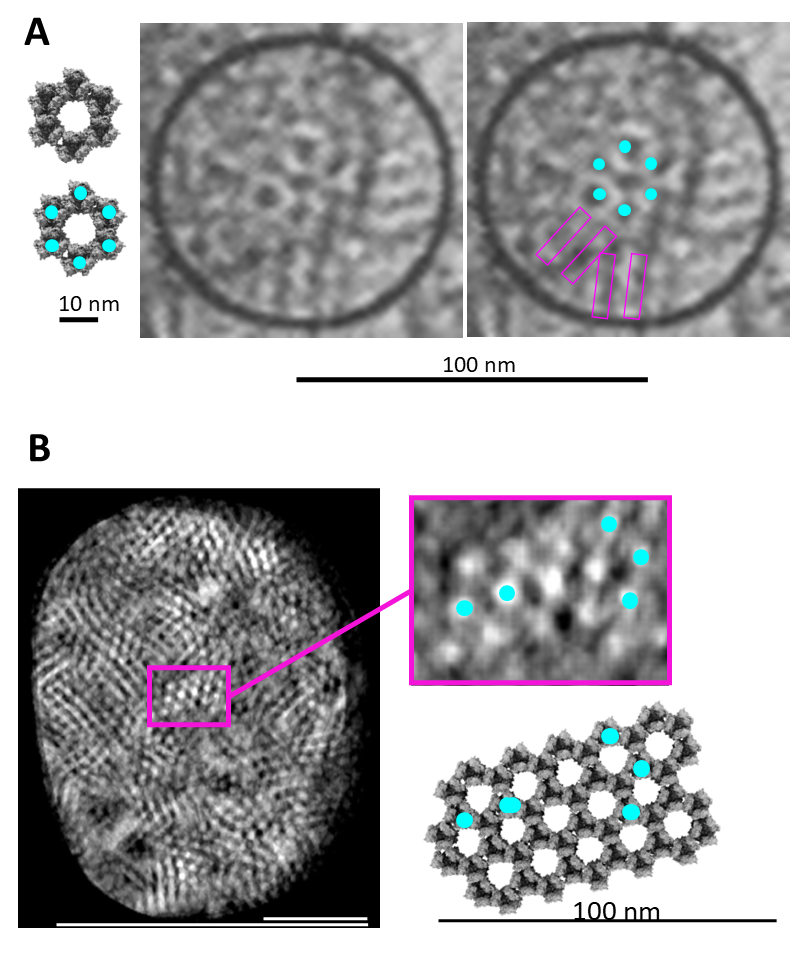
**

**
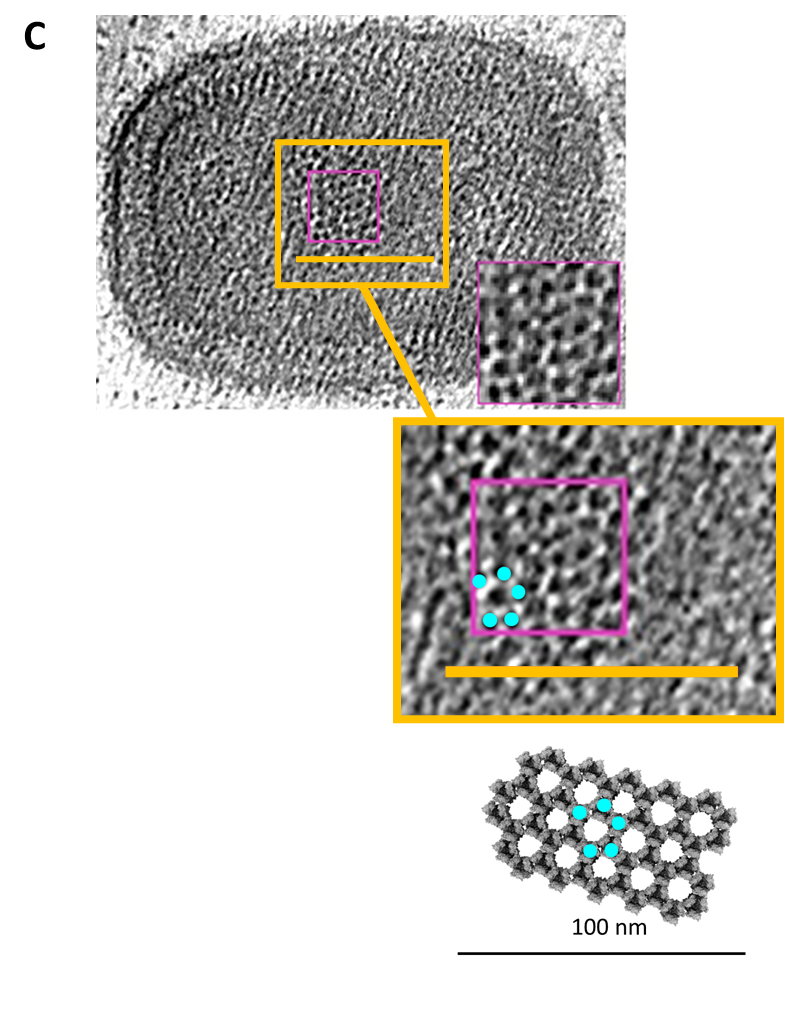
**

**
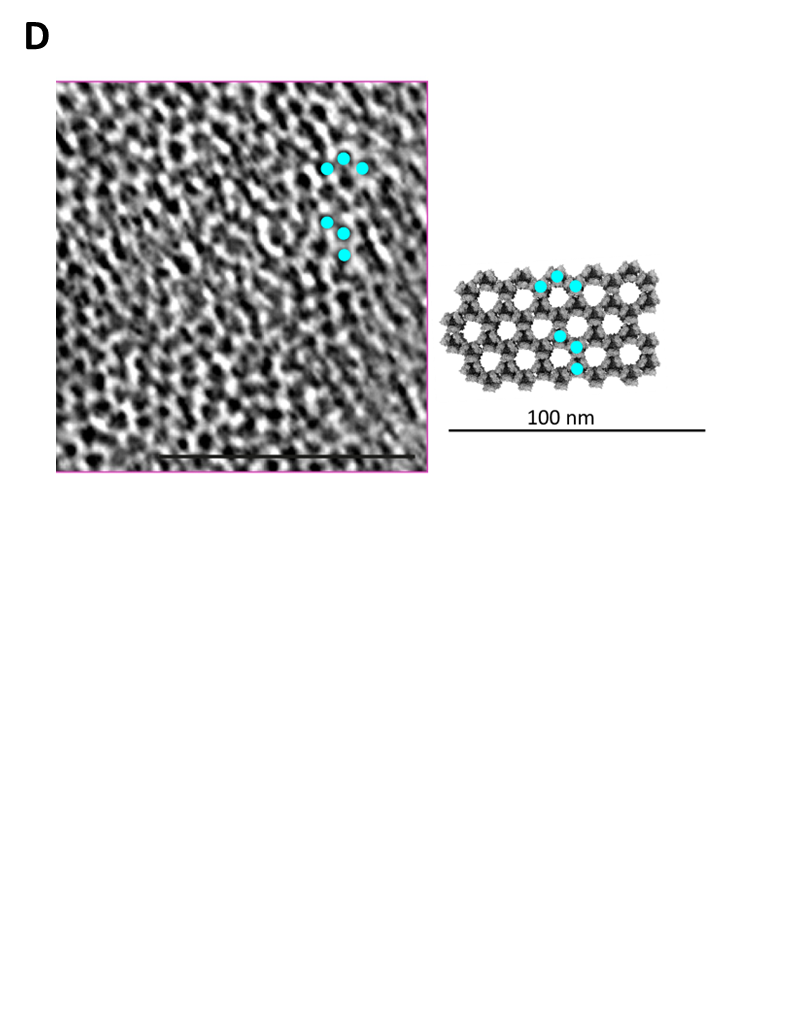
**

**Fig. S14**.

**CryoEM hexagonal features scale with P4a-1 trimer-hexamer.** (**A**) A hexagonal feature on core wall in Fig 7 of ref. (*38*) is comparable in scale to a trimer-hexamer modeled here. Right panel same as left but with pegs outlined magenta. The circular outline was on the original image. The EM images show the same periodicity as cyan puncta of hexagonal features. (**B**) Hexagonal features in Fig 5c of ref. (*41*), comparable in scale to a trimer-hexamer modeled here. Cyan dots are arbitrarily placed in order to register EM images with molecular model lattice. Based on the original figure legend, the colors in this figure are inverted, so white shows regions of higher electron density. (**C and D**) Hexagonal features in Fig 5c and 6b respectively of ref. (*40*), are comparable in scale to the trimer-hexamer modeled here. Scale bars are 100 nm. In panel C, the scale bar was measured and transferred from a different region in Fig 5c (*40)* is shown in orange. Cyan dots were arbitrarily placed to highlight similarities in spacing of hexagonal features.
